# Comparative efficacy and safety of different recommended doses of telitacicept in patients with systemic lupus erythematosus in China: a systematic review and meta-analysis

**DOI:** 10.3389/fimmu.2024.1472292

**Published:** 2025-01-10

**Authors:** Shenglan Gao, Chunlong Yang, Bitang Huang, Lawei Yang, Lu Lu, Huiting Yang, Ting Li, Qingjun Pan

**Affiliations:** ^1^ State Key Laboratory of Quality Research in Chinese Medicine, Macau Institute for Applied Research in Medicine and Health, Macau University of Science and Technology, Macao, Macao SAR, China; ^2^ Clinical Research Center, Affiliated Hospital of Guangdong Medical University, Zhanjiang, China; ^3^ Clinical Research Center, Laboratory Medicine Center, Affiliated Hospital of Guangdong Medical University, Zhanjiang, China; ^4^ Guangdong Provincial Key Laboratory of Autophagy and Major Chronic Non−Communicable Diseases, Affiliated Hospital of Guangdong Medical University, Zhanjiang, China; ^5^ Department of Clinical Laboratory, State Key Laboratory of Respiratory Disease, The First Affiliated Hospital of Guangzhou Medical University, Guangzhou, China

**Keywords:** telitacicept, B lymphocytes, systemic lupus erythematosus, efficacy, safety

## Abstract

**Background:**

Telitacicept, a new biological agent, was approved in China for treating systemic lupus erythematosus (SLE) in 2021. Its optimal dosing for treating SLE remains unclear. Therefore, the aim of this meta-analysis is to evaluate the efficacy and safety of various telitacicept doses in SLE treatment.

**Methods:**

PubMed, EMBASE, Cochrane libraries, Web of science, China National Knowledge Infrastructure (CNKI), VIP, Wanfang, and Sinomed were searched for the controlled trials that studied the efficacy and safety of telitacicept on SLE patients from their initiation to April 30, 2024. The analysis included three randomized controlled trials (RCT) with 606 participants. We used fixed-effects models for meta-analyses and the risk ratios (RRs) and corresponding 95% confidence intervals (CIs) to evaluate the effectiveness and safety. Heterogeneity was assessed and quantified using I^2^.

**Results:**

All telitacicept dosages (80 mg, 160 mg, 240 mg) significantly improved SLE Responder Index 4 (SRI4) responses compared to the control group (RR = 2.20, 95%CI:1.50-3.21, *p* < 0.0001; RR = 2.18, 95%CI: 1.82-2.62, *p* < 0.00001; RR = 2.44, 95%CI: 1.67-3.56, *p* < 0.00001, respectively). The 80 mg, 160 mg, and 240 mg groups also showed better improvement on SELENA-SLE Disease Activity Index (SELENA-SLEDAI) scores (RR = 1.63, 95%CI: 1.23-2.17, *p* = 0.0008; RR = 1.72, 95%CI: 1.45-2.04, *p* < 0.00001; RR = 1.73, 95%CI: 1.30-2.30, *p* = 0.0002, respectively) and Physician Global Assessment (PGA) scores (RR = 1.25, 95%CI: 1.09-1.44, *p* = 0.002; RR = 1.39, 95%CI: 1.25-1.55, *p* < 0.00001; RR = 1.24, 95%CI: 1.09-1.42, *p* = 0.002, respectively). Furthermore, 160 mg group exhibited higher British Isles Lupus Assessment Group (BILAG) score than the control group (RR = 1.11, 95%CI: 1.01-1.22, *p* = 0.03). As for security, 160 mg telitacicept group had higher incidence of adverse events (AEs) than the control group (RR = 1.10, 95%CI: 1.03-1.18, *p* = 0.007).

**Conclusion:**

Telitacicept combined with standard therapy presents potential benefits but there are certain safety concerns with certain dosages of telitacicept, warranting further investigation for optimal dosing strategies in SLE management.

**Systematic review registration:**

INPLASY.COM, identifier INPLASY202440101.

## 1 Introduction

Systemic lupus erythematosus (SLE) is a chronic autoimmune system disorder of complex etiology, marked by the overproduction of autoantibodies that lead to widespread damage across multiple organs and systems (1). The pathogenesis of SLE is influenced by a confluence of genetic susceptibilities, environmental factors, immunological dysregulations, and hormonal imbalances (2–4). Abnormal immune responses are closely associated with SLE pathogenesis, leading to tissue damage by releasing aberrant inflammatory cytokines and abnormal regulation of autoreactive T and B cells (5, 6). The current therapeutic strategies for SLE primarily relied on conventional treatments, glucocorticosteroids and immunosuppressants, which have limited efficacy and can cause several adverse events, such as kidney damage (7, 8). This underscores the need for more effective and safer treatment options.

B lymphocytes play a crucial role in SLE, contributing to disease development by generating autoantibodies, presenting autoantigens, and activating autoreactive T cells, making them key targets for SLE treatment (9, 10). New therapies, particularly those targeting B cells, are revolutionizing SLE management (11). B-cell-activating factor (BAFF) and a proliferation-inducing ligand (APRIL), part of the tumor necrosis factor (TNF) family, are key modulators in B cell biology. They are predominantly expressed by myeloid cells and interact with multiple receptors to influence B cell development, differentiation, and survival (12, 13). BAFF’s interaction with BAFF-receptor (BAFF-R), transmembrane activator and calcium-modulator and cyclophilin ligand interactor (TACI), and B cell maturation antigen (BCMA), is crucial for B cell maturation and plasma cell survival (14). APRIL, similar to BAFF, also supports plasma cells development and survival through TACI and BCMA (15, 16). These factors are also linked to autoimmune diseases like SLE, with higher levels in patients indicating a role in disease progression (16–19). As a result, BAFF and APRIL are promising targets for SLE treatment, with inhibitors such as telitacicept and belimumab already in clinical use. Furthermore, clinical trials are exploring additional agents, reflecting the continuous search for new autoimmune disease treatments.

Telitacicept, also known as Taiai, RC18 or RCT-18, is a novel fully human soluble fusion protein. It is composed of the fragment crystallizable (Fc) domain of human immunoglobulin G1 (IgG1) fused with the extracellular domain of the TACI receptor. This structure allows it to modulate the immune response by lymphocyte receptors (20, 21). In March 2021, it received conditional approval in China for treating active SLE, supported by clinical trials demonstrating its effectiveness and favorable safety (20). Our meta-analysis aims to consolidate data from various controlled trials to evaluate telitacicept’s efficacy and safety in SLE management, providing valuable clinical practice insights.

## 2 Methods

### 2.1 Compliance with Systematic Reviews and Meta-Analyses (PRISMA) guidelines

This systematic review adheres to the Preferred Reporting Items for PRISMA guidelines (22), ensuring a rigorous and transparent approach. The protocol has been registered at INPLASY.COM with the identification number INPLASY202440101.

### 2.2 Inclusion and exclusion criteria for study selection

#### 2.2.1 Inclusion criteria

1. Inclusion of randomized controlled trials (RCTs) that evaluate the efficacy and safety of telitacicept in patients with SLE. These studies provide the most robust evidence for assessing treatment outcomes.
2. Focus on patients diagnosed with SLE.

#### 2.2.2 Exclusion criteria

1. Lack of control group to ensure that the efficacy of telitacicept can be compared against a standard of care or placebo.
2. Review articles as they do not contain original data and are not primary sources of evidence.
3. No related outcomes to maintain the focus and relevance of our systematic review and meta-analysis.
4. Duplicate publications to avoid redundancy in data analysis and to ensure the integrity of our findings.
5. Ongoing trials to ensure that only studies with complete data and final outcomes are included in our analysis.
6. Unpublished material including non-peer-reviewed articles to ensure that all included studies have undergone rigorous peer review and meet the standards of scientific credibility.

### 2.3 Participant characteristics

All participants in the included studies were diagnosed with active SLE. Detailly, SLE patients aged 18 to 65 years who met the 1997 American College of Rheumatology criteria for SLE (23, 24) and receive stable standard therapy with positive ANA and/or anti-dsDNA and a SELENA-SLE Disease Activity Index (SELENA-SLEDAI) score ≥8.

### 2.4 Intervention and comparator

The intervention involved the use of telitacicept, either as monotherapy or in combination with standard treatment protocols. The control group received either placebo or alternative immunosuppressive medications.

### 2.5 Outcome measures

The primary endpoint is the SLE Responder Index 4 (SRI4), the secondary outcomes include a reduction more than 4 point from baseline in SELENA-SLEDAI score, no worsening in Physician Global Assessment (PGA) score, indicated by an increase less than 0.30 points from baseline, no new 1A/1B British Isles Lupus Assessment Group (BILAG 1A/1B) organ domain scores compared with baseline the incidence of adverse events (AEs) and serious adverse events (SAEs).

### 2.6 Search strategy

A comprehensive search was conducted across multiple databases, including PubMed, EMBASE, Cochrane libraries, Web of Science, China National Knowledge Infrastructure (CNKI), VIP, Wanfang, and Sinomed up to April 30, 2024. The search utilized a combination of keywords and index terms related to telitacicept and SLE. Moreover, all the screened and included articles in English or Chinese.

### 2.7 Data extraction and analysis

#### 2.7.1 Studies selection

Two researchers independently screened articles and extracted relevant data, with disagreements resolved by a third independent critic.

#### 2.7.2 Data extraction

Extracted data included general study information, participant characteristics, intervention details, and outcomes.

#### 2.7.3 Risk of bias assessment

The risk of bias in the included RCTs was appraised using the Cochrane Collaboration’s “risk of bias” tool, following the guidelines outlined in the Cochrane manual version 5.1.0. This evaluation encompassed key aspects such as the generation of random sequences, concealment of allocation, implementation of blinding, handling of incomplete data, and the integrity of reported results, as well as the potential for other biases. The studies were categorized into “high,” “unclear,” or “low” risk of bias.

#### 2.7.4 Statistical analysis for meta-analysis

The meta-analysis was conducted using Review Manager 5.4, focusing on the comparative efficacy and safety of different telitacicept dosages against control treatments. We calculated the weighted mean difference (WMD) and 95% confidence intervals (CIs) to quantify the impact of telitacicept across various concentrations. For dichotomous outcomes, the risk ratio (RR) with 95% CIs was determined, representing the ratio of event occurrence probabilities between the treatment and control groups. Statistical heterogeneity among studies was assessed using the Chi-square test and the I^2^ statistic. In line with the recommendation by Murad et al. for a meta-analysis when the number of the included studies is less than five, a fixed-effects model was applied to pool the RRs for both primary and secondary endpoints (25).

## 3 Results

### 3.1 Study characteristics

**Figure 1:**
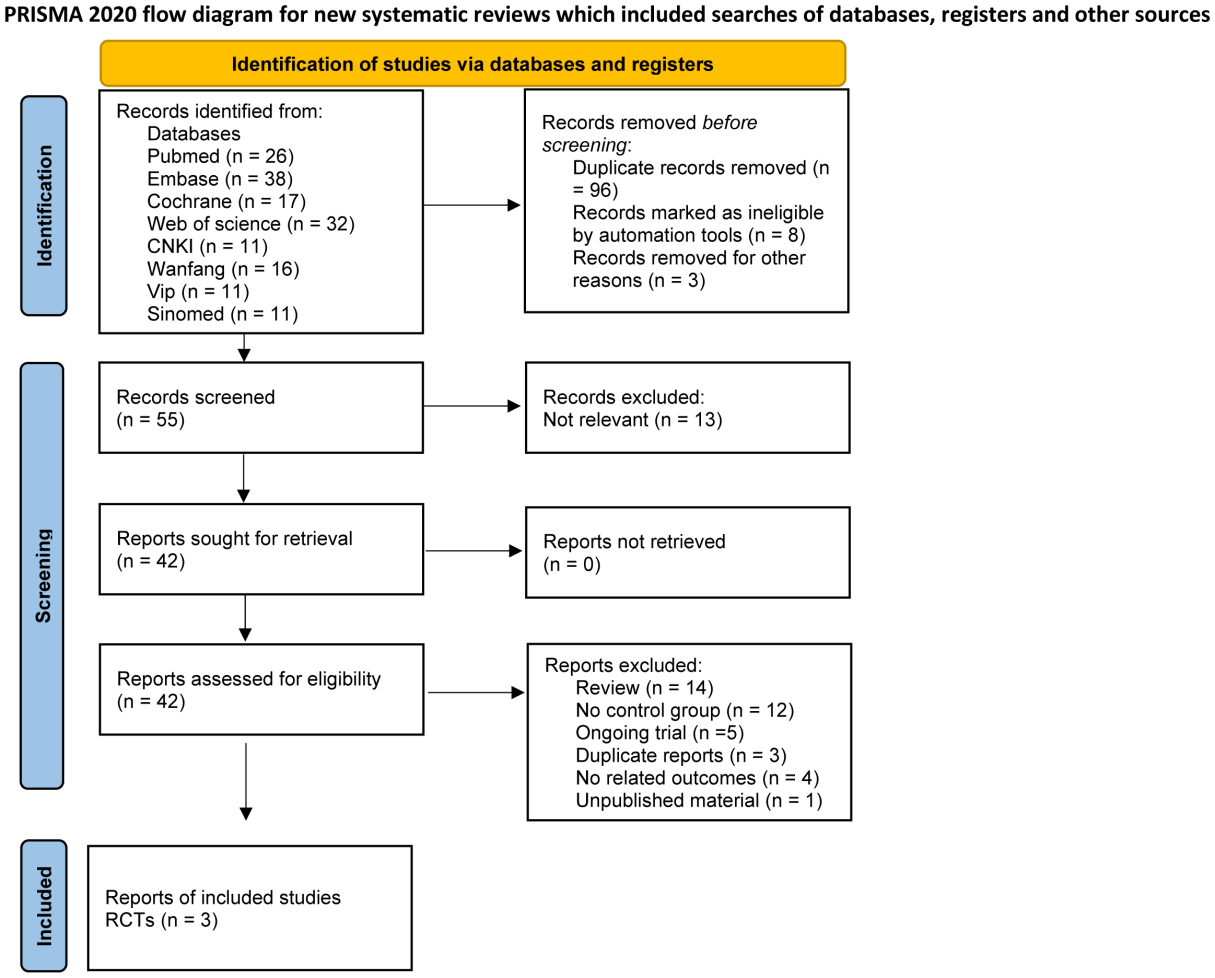
PRISMA 2020 flow diagram.

Our search extracted 162 studies and abstracts. Following a meticulous review, 96 duplicates were identified and excluded. An additional 8 records were removed due to ineligibility as determined by automated tools, and 3 were excluded for unrelated reasons such as drug registration certificates, expert consensus documents, and medical insurance catalogs. This process resulted in 55 records for initial screening, with 42 full reports assessed for eligibility. Ultimately, 39 studies were excluded for not meeting the inclusion criteria, leading to the inclusion of three RCTs articles (26–28) in this meta-analysis (**Figure 1**). The three included studies reported data on dosages, encompassing low, moderate, and high doses, and involved a total of 606 participants.

The characteristics of the included studies are detailed in **Table 1**. All three studies provided data on the SRI4, adverse events, and serious adverse events in the telitacicept groups and the control group. Three studies reported the reduction in SELENA-SLEDAI scores and the increase of PGA scores. Additionally, two studies documented the BILAG 1A/1B scores among participants.

**Table 1:** Characteristics of the studies included in the meta-analysis.

Source	Study Design	Country	Follow-up, Weeks	Interventions	Numbers	Outcomes
Wu 2024	RCT	China	48	RC18 80 mg/kg	62	①②③④⑤⑥
				RC18 160 mg/kg	63	
				RC18 240 mg/kg	62	
				Placebo	62	
Wang 2023	RCT	China	52	RC18 160 mg/kg	167	①②③⑤⑥
				Placebo	168	
Jiang 2020	RCT	China	48	RC18 80 mg/kg	3	①②③④⑤⑥
				RC18 160 mg/kg	7	
				RC18 240 mg/kg	7	
				Placebo	5	

① SRI4, ② SELENA-SLEDAI score, ③ PGA score, ④ BILAG domain, ⑤ adverse events and ⑥ serious adverse events.

### 3.2 Risk of bias assessment

**Figure 2:**
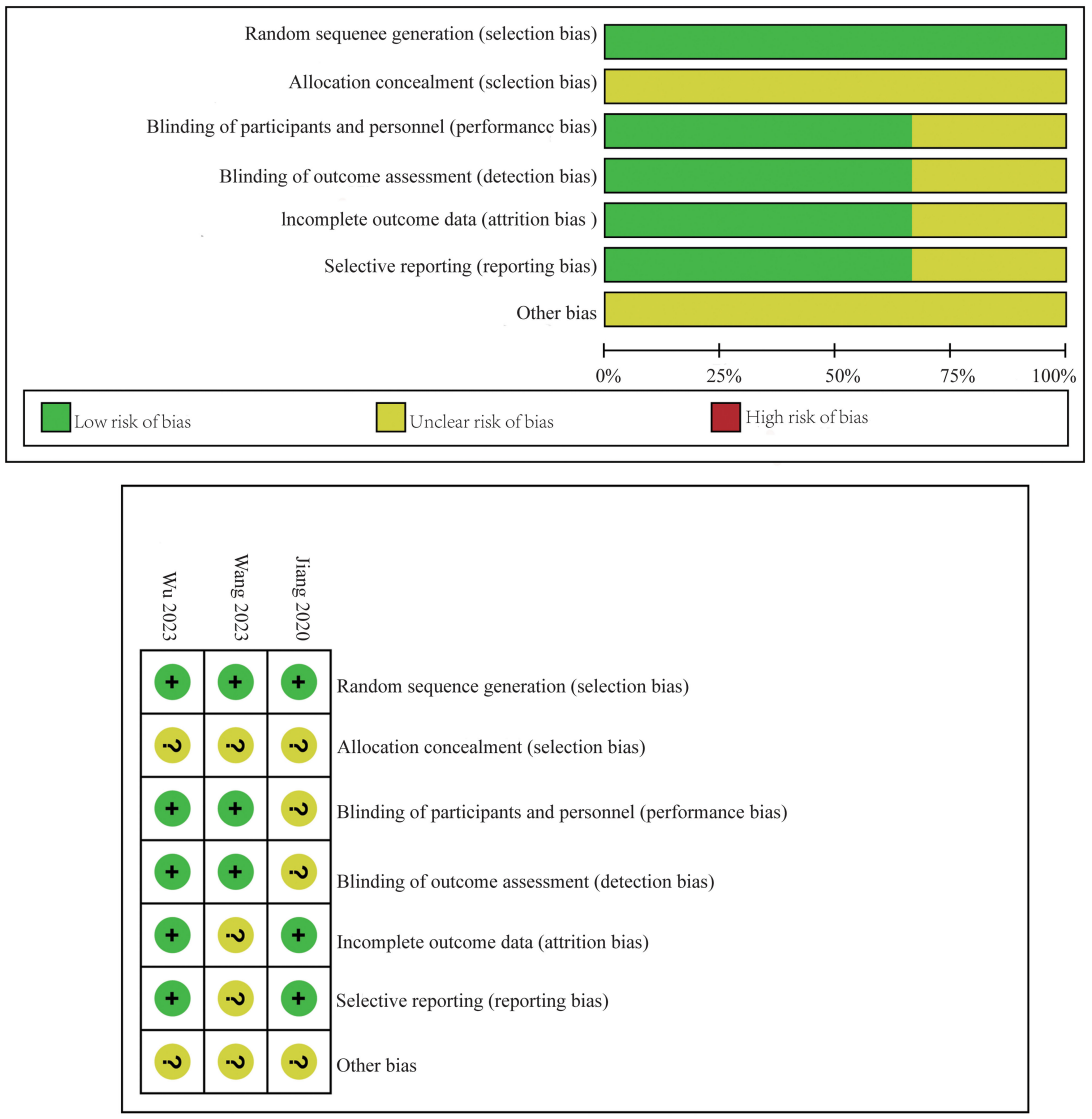
Risk of bias assessment.

The risk of bias for the RCTs included in this meta-analysis was detailed in **Figure 2**. This assessment was conducted using the established criteria from the Cochrane Collaboration. The evaluation aimed to identify any potential sources of bias that could affect the reliability of the findings.

### 3.3 Effects of telitacicept on SRI4 response

**Figure 3:**
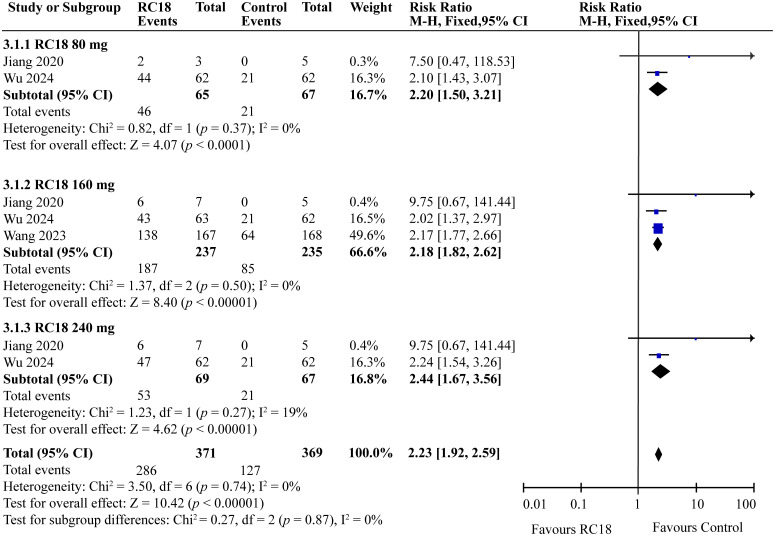
Meta-analysis of SRI4 response.

Three articles provided the SRI4 in the telitacicept group and the control group. A total of 606 patients were involved, with 371 in the telitacicept group and 235 in the control group. The results of the meta-analysis showed that the percentage of achieving SRI4 responses in 80 mg telitacicept group (RR = 2.20, 95%CI:1.50-3.21, *p* < 0.0001), 160 mg telitacicept group (RR = 2.18, 95%CI: 1.82-2.62, *p* < 0.00001), 240mg telitacicept group (RR = 2.44, 95%CI: 1.67-3.56, *p* < 0.00001) were significantly higher than the control group (**Figure 3**). In all dosage groups, the rate of achieving SRI4 responses was markedly superior to that of the control group.

### 3.4 Effects of telitacicept on SELENA-SLEDAI score

**Figure 4:**
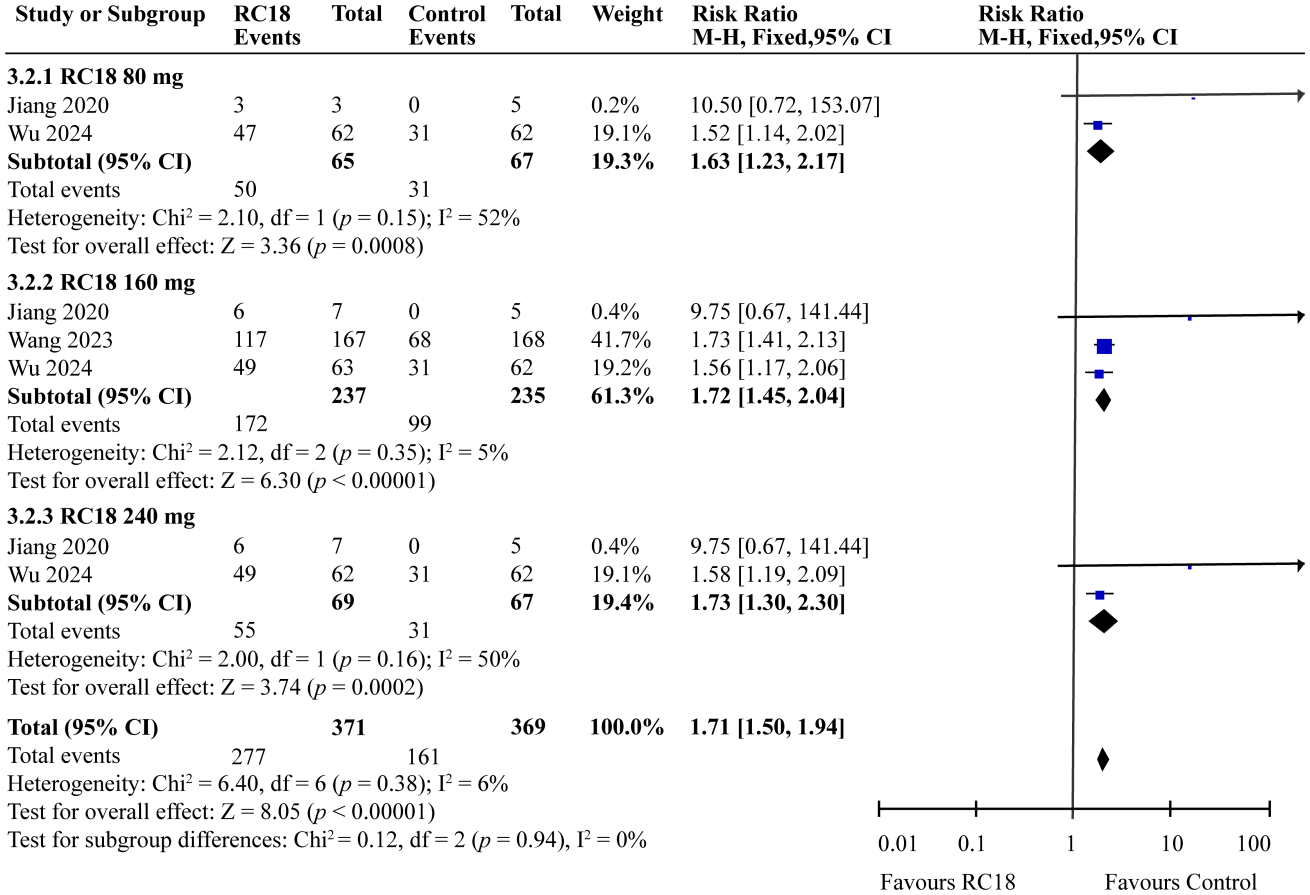
Meta-analysis of SELENA-SLEDAI.

Our meta-analysis incorporated data from all articles that evaluated the reduction of at least 4 points in the SELENA-SLEDAI score. The results of the meta-analysis showed that SLEDAI score also exhibited better effects in 80 mg telitacicept group (RR = 1.63, 95%CI: 1.23-2.17, *p* = 0.0008). 160 mg telitacicept group (RR = 1.72, 95%CI: 1.45-2.04, *p* < 0.00001), 240 mg telitacicept group (RR = 1.73, 95%CI: 1.30-2.30, *p* = 0.0002) than the control group (**Figure 4**), reflecting a significant benefit over the control group in terms of SLEDAI score reduction.

### 3.5 Effects of telitacicept on PGA score

**Figure 5:**
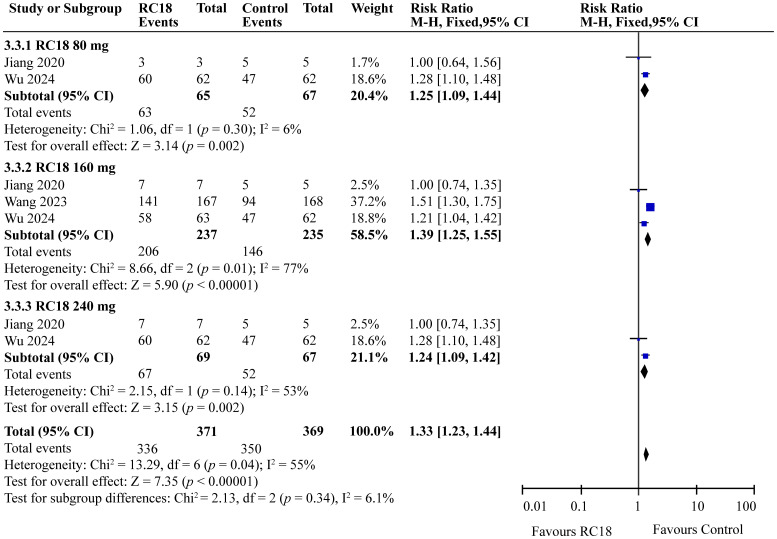
Meta-analysis of PGA score.

In our meta-analysis, we examined the effects of telitacicept on PGA scores, as reported in three articles. The meta-analysis revealed significant improvements in the 80 mg telitacicept group (RR = 1.25, 95%CI: 1.09-1.44, *p* = 0.002). Similarly, 160 mg telitacicept group and 240 mg telitacicept group also showed significant improvement (RR = 1.39, 95%CI: 1.25-1.55, *p* < 0.00001, RR = 1.24, 95%CI: 1.09-1.42, *p* = 0.002, respectively). All dosages demonstrated a better performance in improving PGA scores compared to the control group (**Figure 5**).

### 3.6 Effects of telitacicept on BILAG score

**Figure 6:**
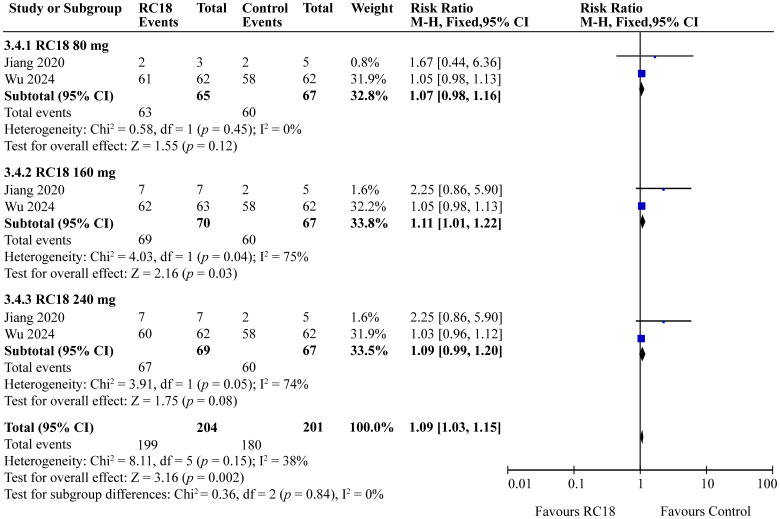
Meta-analysis of BILAG domain.

The meta-analysis incorporated findings from two articles that reported the percentages of patients with no worsening in BILAG scores. The study population comprised a total of 606 patients, with 204 in the telitacicept group and 67 in the control group. The 160 mg telitacicept group showed statistically significant improvement in BILAG response compared to the control group (RR = 1.11, 95%CI: 1.01-1.22, *p* = 0.03). This suggests that 160 mg telitacicept may be effective in maintaining stability in BILAG scores. In contrast, the 80 mg and 240 mg telitacicept groups did not demonstrate significant differences in BILAG response compared to the control group (all *p* > 0.05). This finding indicates that the effect of telitacicept on BILAG scores may be dosage-dependent, and the 160 mg dosage might be particularly effective in preventing worsening of BILAG scores (**Figure 6**).

### 3.7 Effect of telitacicept on safety

**Figure 7:**
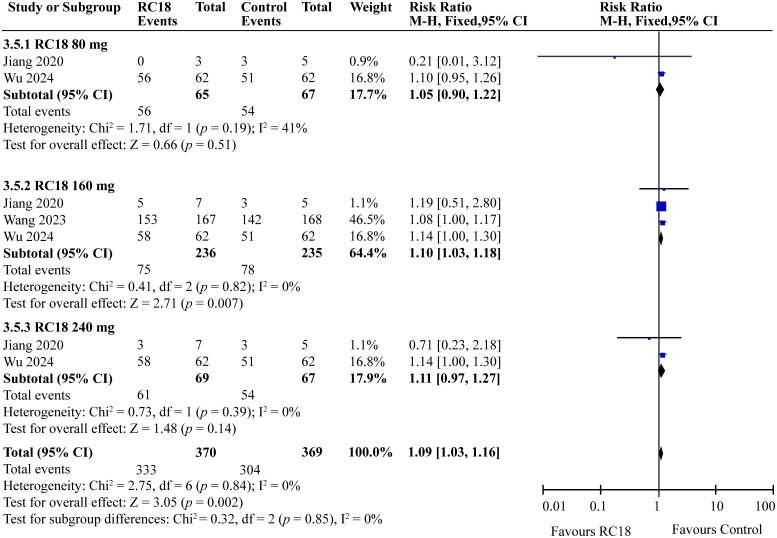
Meta-analysis of adverse effects.

**Figure 8:**
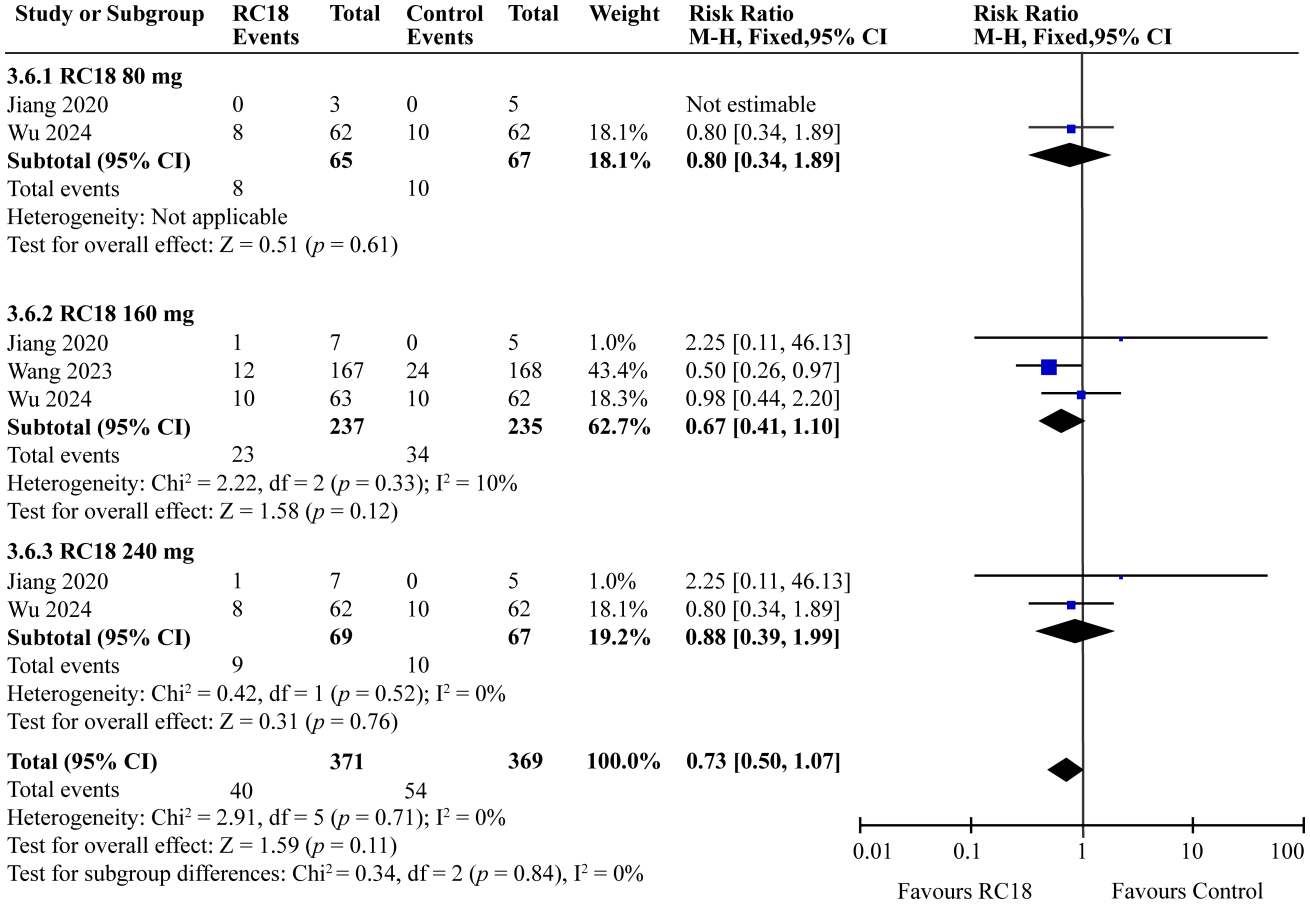
Meta-analysis of serious adverse events.

In the comprehensive analysis of safety outcomes, all studies reported the incidence of adverse events (AEs) associated with telitacicept treatment. The majority of these AEs were categorized as mild to moderate infections, predominantly upper respiratory tract infections and urinary tract infections. The meta-analysis revealed that the incidence of AEs in the 80 mg and 240 mg telitacicept group was comparable to that of the control group, indicating a similar safety profile between the treatment and control cohorts (all *p* > 0.05) . However, 160 mg telitacicept group showed higher incidence of AEs than the control group (RR = 1.10, 95%CI: 1.03-1.18, p = 0.007) (**Figure 7**). Furthermore, the incidence of serious adverse events (SAEs) was also evaluated across all studies. SAEs included conditions such as tuberculosis and severe infections that necessitated hospitalization or were potentially life-threatening. The results of the meta-analysis indicated no significant difference in the incidence of SAEs between the treatment groups receiving telitacicept and the control group (all *p* > 0.05) (**Figure 8**). This finding suggests that different doses of telitacicept appear to have different risks of adverse events compared to standard treatments.

## 4 Discussion

The standard clinical approach to the treatment of SLE encompasses a range of therapies, including immunomodulatory agents, glucocorticoids, non-biologic immunosuppressives, and more recently, biologic agents (29). The latter have gained prominence due to their targeted nature, offering improved efficacy and safety profiles. B lymphocytes and their associated signaling pathways are recognized as integral to SLE pathogenesis (30, 31). Rituximab, a chimeric anti-CD20 monoclonal antibody, and belimumab, which targets B-lymphocyte stimulator, are among the biologic agents used in SLE treatment. While rituximab is recommended for moderate to severe SLE, its efficacy is a subject of debate and it is associated with several adverse effects. Belimumab, approved in 2011 for active SLE, has demonstrated disease progression benefits and tolerability but still results in mild to moderate adverse events (29). Telitacicept represents a novel class of biological agents, a humanized, soluble recombinant TACI-Fc fusion protein that neutralizes both BAFF and APRIL. By antagonizing their interaction with B lymphocytes, telitacicept suppresses B cell-mediated autoimmune responses (14, 32). Clinical trials have shown promising results, with phase IIb trials and phase III study indicating improved SRI4 rates and a favorable safety profile (26, 27)

Our meta-analysis has investigated the effects of varying concentrations of telitacicept on SLE efficacy and safety. The results suggest that all tested doses (80 mg, 160 mg, and 240 mg) significantly improve SRI4 responses, SELENASLEDAI score and PGA score in SLE patients. Dose subgroup analysis indicates a potential for increased efficacy with higher telitacicept concentrations. The results of the safety analysis indicate that the 80 mg and 240 mg doses of telitacicept have similar safety profiles to the control group, with no significant differences in adverse events. However, the 160 mg dose showed a significant increase in adverse events compared to the control group. To balance the efficacy and safety of telitacicept, several strategies can be considered, such as individualized dosing and monitoring and managing adverse reactions. Specifically, start with a lower dose (e.g., 80 mg) for patients with mild conditions or poor drug tolerance. Adjust the dose based on therapeutic response and adverse reactions, increasing it if needed for better efficacy or decreasing it if adverse effects occur. Regularly monitor liver function and blood indices to promptly identify adverse reactions. In cases of significant liver enzyme elevations or severe infections, consider adjusting the dose or implementing appropriate therapeutic interventions.

A real life observational study reported that after 4-45 weeks’ administration of telitacicept, the SRI4 response rate of SLE patients were significantly increased and the glucocorticoid and immunosuppressive drugs were obviously reduced, indicating a potential treatment option of telitacicept for patients with SLE (33). Huang et al. evaluated the safety and efficacy of telitacicept in managing patients with lupus nephritis (LN). With an SRI4 response rate of 86.67% and significant reductions in SLEDAI scores, which supports the effectiveness of telitacicept in reducing disease severity in LN patients (34). Moreover, this case series reported by Fan et al. offers detailed insights into how telitacicept has been used in patients who did not respond to conventional treatments, highlighting its potential as a therapeutic option for refractory cases (35). Jin and colleagues conducted a multicenter study that examined the efficacy and safety of telitacicept in everyday clinical practice and they found favorable outcomes in patients with active SLE, with significant decreases in serum IgA, IgG, and IgM levels and improvements in renal and hematological manifestations of the disease. This study provides additional evidence of telitacicept’s efficacy and safety in patients with active SLE, highlighting its potential in improving renal and hematological abnormalities (36).

We have identified studies that evaluate the efficacy and safety of telitacicept in comparison with other biologics, such as belimumab. A recent study compared telitacicept and belimumab in patients with SLE, highlighting the therapeutic efficacy of telitacicept. After receiving telitacicept treatment, patients with active SLE demonstrated a higher SRI4 response rate, more significant reductions in both the SLEDAI-2K and PGA scores, and a lower rate of adverse events at the 24 weeks compared to those treated with belimumab (37). A case report showed by Huang et al. (38) and case series study conducted by Fan et al. (35) both reported that SLE patients who accepted telitacicept treatment after failed treatment with belimumab showed significant improvement of disease activity, revealing that telitacicept may be a promising drug for SLE treatment. This dual targeting provides a unique framework for differentiating treatments and has been shown to inhibit the formation of plasma cells and the secretion of autoantibodies.

It is important to acknowledge the limitations of this study. As a relatively new biologic, there is a paucity of clinical studies on telitacicept for SLE treatment. The need for large sample sizes and long-term follow-up, and serum pharmacodynamic biomarkers including IgG, IgA and IgM, complement components C3 and C4 as well as CD19^+^ cell counts in clinical studies to assess drug efficacy and safety is well recognized. Many studies are still in progress, and their results are eagerly awaited to further validate these findings. Additionally, the inconsistent follow-up times across studies may affect the statistical power and reliability of the results. Lastly, while different concentrations of telitacicept were compared to a control group, a more detailed quantitative analysis of the differences between concentrations is needed. Despite these limitations, telitacicept has demonstrated satisfactory results in the treatment of RCT, showing great potential and promising application in this field. The ongoing clinical trials and future research will provide a more comprehensive understanding of telitacicept’s role in SLE management.

## 5 Conclusion

The findings of this meta-analysis provide compelling evidence that the combination of telitacicept with standard therapy offers superior efficacy in the treatment of SLE compared to placebo or other biologics such as belimumab, when used in conjunction with standard treatment protocols. This conclusion is drawn from a rigorous synthesis of data from multiple clinical studies, demonstrating the potential of telitacicept as a valuable addition to the therapeutic arsenal for SLE.

## Data Availability

The original contributions presented in the study are included in the article/supplementary material. Further inquiries can be directed to the corresponding authors.
